# Editorial

**Published:** 1841-03-06

**Authors:** 


THE MEDICAL EXAMINER.

PHILADELPHIA, MARCH 6, 1841.

### PROFESSIONAL EMOLUMENTS.

We remember to have heard the opinion advanced by a veteran confrère, that a greater amount was expended upon the education of physicians, than in the aggregate they received from the public. Startling as this assertion may seem, it is nearer the truth than is generally supposed. It is a fact, seriously to be pondered on, that professional compensation is very disproportionate to the physical and intellectual labour expended in earning it. The road to wealth is not often smoothed by the doctor's gig. While, in this country, almost every other avocation, pursued steadily and with industry, holds out a fair prospect of accumulation, the physician in general toils on, fortunate if he can secure an income adequate to support his position in society, dependent on and expiring with his own exertions. He is indeed rewarded with a desirable position in society, and the self-approbation which accompanies the honourable discharge of the duties of a noble calling. But he has a right to look to more—to the attainment of those objects which most men prize, ease and comfort for the decline of life, and competence for the families which they rear. Why are these objects generally beyond the reach of physicians? Why, in our favoured country, do they, almost alone, among the intelligent industrious, fail to secure that summum bonum, wealth, in this thriving age the great object of human effort? We think the cause lies very much with themselves. In this, our own city, the American metropolis of medicine, we are satisfied that the profession can by proper concert of action, greatly advance the general standing and individual prosperity of its members.

Might we venture to offer suggestions to those who occupy the foremost ranks, we would say—concentrate your business, and exact (what will be cheerfully conceded) higher rates of compensation. Thus, with greater comfort to yourselves, with less expenditure of vital energy, of which many of you are recklessly lavish, your patients will receive more attention, your own incomes be improved, and your juniors be spared the long years of hope deferred. We could point to more than one, eminent among us, whose desire to enlarge their business is not measured by their ability to attend to it with justice, if not to their patients, certainly to themselves; to more than one who, from a mistaken liberality to patients, commit great unfairness to their brethren. It is this very carelessness of compensation, too, that often swells the business of a popular practitioner to the inordinate amount which places him in the position of the dog in the fable. We repeat, that such would increase their own comfort, their own and the general prosperity by working less at higher rates. Most of the profession here will feel the justice of these remarks.

It is indisputably the duty of *every* practitioner strictly to adhere, not so much to a fixed tariff of fees, as to a scale of compensation due to the dignity of his profession, and a proper estimate of his own usefulness. In cases appropriate for charity, let him remit according to his discretion; but let him in no instance undervalue, even nominally, services of the highest grade which man can render to man. Doubtless, the same remuneration cannot be asked from the poor as from the opulent, but every physician owes to his profession, if not to himself, to maintain a proper standard of remuneration, which he can always at least assert.

The gratuitous services rendered to public charities form a proper topic of remark, while discussing the general subject. This point is, however, of such peculiar delicacy, that we prefer deferring it for special notice. We advert to the whole matter, it is perhaps hardly necessary to add, from a sense of editorial duty, choosing, even at the risk of being misunderstood, to uphold what we think the interests and dignity of the profession. These objects, as well as the advancement of medical science, come within the scope of a journal. We invite the attention of correspondents to the subject.

